# End-Users and Caregivers’ Involvement in Health Interventional Research Carried Out in Geriatric Facilities: A Systematic Review

**DOI:** 10.3390/ijerph16162812

**Published:** 2019-08-07

**Authors:** Mathieu Ahouah, Monique Rothan-Tondeur

**Affiliations:** 1 University Paris 13, Sorbonne Paris Cite, Nursing Sciences Research chair, Laboratory Educations and Health Practices (LEPS), (EA 3412), UFR SMBH, F-93017 Bobigny, France; 2 Assistance Publique Hôpitaux de Paris (AP HP), Nursing sciences Research Chair, 75004 Paris, France

**Keywords:** geriatric, involvement, health interventions

## Abstract

Public involvement (PI) is of great interest. However, little is known about this topic in the design, development, and/or implementation of health interventions in geriatric facilities. This study aimed to provide a critical overview of the involvement of caregivers and end-users in interventions in these facilities, based on Rifkin’s analytical framework. This systematic review, supplemented by a questionnaire to the corresponding authors, covered non-drug intervention reports targeting nurses, doctors, residents, and their relatives. Articles were published in Pubmed, Medline, Scopus, and Cinahl, from January 2016 to April 2018. Ninety-seven articles were included. The review shows a low level or partial PI in geriatric facilities where it exists. These results are further supported by the authors’ responses to the questionnaire. PI remains uncommon in geriatric institutions and consists of a consumerist model, suggesting the need for improved practices. More efforts are needed to experiment with recommendations to meet the challenges of PI and enhance the public ownership of interventions. The protocol was registered on Prospero under the number CRD42018098504.

## 1. Introduction

The current aging era, resulting from the improvement of health-related quality of life [1] and global care, as well as living conditions [2], will contribute to increased research targeting elderly population facilities. This increased aging is associated with increased health issues, such as chronic diseases and falls, as well as undernutrition or drugs management, particularly in geriatric institutions where elderly people are commonly institutionalized [3]. Residents of nursing homes are three times more likely to fall than a similar group in the general population [4], while malnutrition is frequent in nursing homes [5]. In addition, low back pain is frequent in nursing homes where patient handling is common [6]. Therefore, closely involving the public of geriatric facilities in research is of great importance. This involvement could help to better define the scope of research and improve factors, such as sustainability [7], dignity, self-respect, responsibility, social identity, and empowerment of research participants [8]. According to some researchers, involvement would balance the power between researchers and research participants [9]. It is ethically recommended to involve participants in the design of health interventions. Furthermore, many research projects are funded by governments through taxes [9] that are supported by the general population.

Rifkin and her colleagues proposed an analytical framework in the form of a pentagram [7,10]. They defined involvement as “the social process by which specific groups with shared needs living in a defined geographical area actively pursue the identification of their needs, make decisions, and establish mechanisms to meet their needs”. According to Rifkin et al., this process is characterized by five different factors that can be used as a proxy for involvement in an intervention. These factors are as follows: Needs assessment, leadership, organization, resource mobilization, and management.

Despite a great interest for public involvement in research [11], little is known about how researchers are implementing it. Other than our knowledge by screening PUBMED, no studies have been conducted to describe public involvement in geriatric facilities in the design, development, and/or implementation of interventions in these settings.

Therefore, this study was undertaken to support the public involvement process in geriatric facilities in order to strengthen the design of a planned intervention in nursing homes. We aimed to provide an overview of the involvement of nurses, physicians, residents, and their families in research describing non-drug interventions or behavioral or practice-based interventions. This overview focused on the level and implementation of the PI by applying the Rifkin analytical framework [12]. In this study, we used public involvement as public participation.

## 2. Method

This study consisted of a systematic review of the literature supplemented by a questionnaire to the corresponding authors of the articles included in the review. A protocol was drafted and registered on PROSPERO, the International Registry of Systematic Reviews (CRD42018098504).

### 2.1. The Systematic Review

#### 2.1.1. Search Strategy, Criteria, and Approach

Four databases considered relevant with regards to our research question were screened to identify articles written in English or French, published from 1 January, 2016 to 30 April, 2018. This time period was chosen in order to obtain better insights into the most recent interventions targeting residents, their relatives, and health professionals (nurses and doctors), and according to the date of the beginning of this review. This time period was also used to inform an upcoming interventional study in nursing homes. The four selected databases were MEDLINE, SCOPUS, WEB OF SCIENCE, and CINHAL. Prior to the search equation, the authors screened PUBMED to analyze reviews of systematic review dealing with interventions implemented in long-term facilities. The purpose of this preliminary work was to identify relevant keywords for designing the combination query (Table S1). Then, a combination of keywords was used to query the databases and identify eligible relevant studies that focus on interventions targeting knowledge, practices, or behaviors in long-term care facilities [13,14] (Table S1).

Articles eligible for the present study met the following study design criteria: Single or cluster randomized trials, before–after studies or time series, cross sectional studies, case report or qualitative studies, and mixed studies approach. Literature reviews, drugs clinical researches, and diagnostic and prognostic tests were not included in this study. Clinical, diagnostic, and prognostic studies were too specific to be included and were out of scope of the present work as we did not aim to implement a review of systematic review studies. In the perspective of a planned intervention by our research team, all interventions in the articles included in this study targeted nurses, physicians, residents, and their families.

We imported all the references found in the four aforementioned databases to ZOTERO software version 5.0.55.1 (Center for History and New Media, Fairfax, VA, USA) to identify duplicate articles and remove them. The different steps of screening and analyses were conducted by the two authors with discussion in case of discrepancies. The next steps consisted of checking the abstracts, the language of the publication, the type of intervention involved, and the participants included in these interventions, as well as the study designs. When a summary of an article fulfilled all the criteria described above, the full article was retained for a full-text assessment. Finally, public involvement in interventions of full texts meeting all criteria was analyzed.

#### 2.1.2. Assessment of Involvement in the Full Texts

The assessment of involvement consisted of three steps based on a score provided by the analytical framework of Rifkin and associates [7,10]. The score ranges from a minimum of 5 (33%) up to a maximum of 15 (100%) for an article. The score was the sum of the notation of each item (Table 1).

The first step in the appraisal was to classify an intervention as involving the public concerned. Interventions involving participants were defined as those with a score over 5.

The second step was the assessment of the level of this involvement. A score of 15 (100%) was equivalent to full involvement. From a score of 6 (40%) to 14 (93%), involvement was considered weak or moderate. Finally, we described the content of this involvement according to the five established items (Table 1) of the analytical framework of Rifkin. Both authors extracted this content manually using an Excel sheet by reading the full text of each included article. The content consisted of qualitative data, such as the type of intervention and its content, to ensure that it met all criteria. Discussions were held in case of discrepancies. Then, authors looked for the roles of the participants in the intervention (are they involved in the different elements described by Rifkin? How are decisions about the intervention made? Have the participants contributed to the means put in place to promote feasibility?). In addition, the two authors assigned a rating to each element of “Rifkin”, also discussing discrepancies when they occurred. We assigned one score per article. In case of a disagreement, we discussed and assigned a score by consensus.

#### 2.1.3. Quality Assessment of Included Articles

Public involvement is a research process. Therefore, our purpose was not to assess the impact of the interventions described in the included articles. The quality of our review was assessed using an AMSTAR grid and MMAT (Mixed Methods Appraisal Tools version 2018) [15] for the studies included in this review (Table S2).

### 2.2. The Questionnaire

An open-ended email questionnaire exploring the five items of Rifkin’s analytical framework was distributed to the corresponding authors of the articles included in the review analysis from 15 June, 2018 to 30 June, 2018. The corresponding authors were invited to explain how they involved the beneficiaries in the design and/or management of their intervention. The questionnaire was also used to check the type of participants identified during the systematic review and their role in the design of the intervention.

Prior to sending the questionnaires, the email addresses of the corresponding authors were extracted from the included articles. A response to the questionnaire was expected seven days after the first emails were sent. However, when a correspondent’s address was not valid during the first mailing, the researcher’s new institutions searched for his new correspondence address. This was carried out through internet and social network search engines (Google, LinkedIn). In addition, a second batch of questionnaires was sent to newfound addresses and valid email addresses for which the authors did not provide a response after the first mailing. Finally, seven days after the second mailing, the corresponding author was definitively considered by the authors of the present review as a non-respondent in the event of a non-response to the questionnaire. This short time period was to avoid a delay in the reporting of the review.

## 3. Results

Our results were divided into two main parts.

### 3.1. The Systematic Review

#### 3.1.1. Characteristics of Included Articles

**Figure 1:**
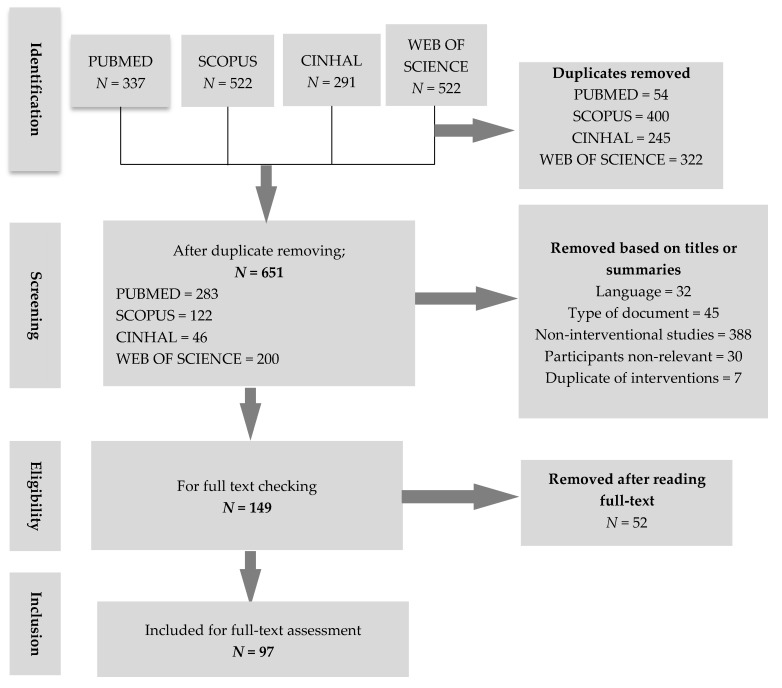
The inclusion process.

**Figure 2:**
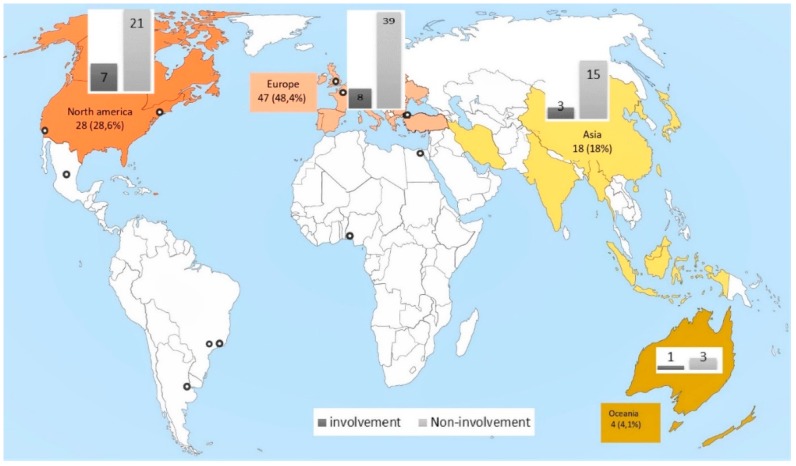
Distribution of included articles and interventions with involvement according to continent of publication.

We summarized the whole article inclusion process in Figure 1. Ninety-seven full-text articles published in 61 different journals in English (100%) were analyzed. The number of articles per journal varied from 1 to 6. The articles included were from four continents and described interventions that had taken place in 28 different countries (Figure 2).

Four main different study designs were identified in the articles included in this review. They were listed as follows: 27 simple randomized trials (26.8%), 21 randomized cluster trials (21.6%), 18 non-randomized trials (18.6%) and 31 quasi-experimental studies (32.0%).

The articles dealt with four major topics. These were interventions about quality of life (17/97), which accounted for 17.5% of the entire articles, while the prescription of medication, falls, and depression accounted each for 6.2%. Education alone was implemented in 44.3% of the interventions and was associated to another type of intervention in 10.3% of the articles analyzed. Moreover, the interventions consisted of physical activities in ten articles (10.3%) and 21.6% of the interventions consisted of participants’ training.

A single category of participants (physicians, nurses, family members, or residents) was targeted in the interventions in 80.4% of the articles analyzed (78/97). These interventions were described as follows: Participants were elderly residents in 37.1% of the articles (36/97), respectively, family members or physicians in one intervention and nurses in 41.2% (40/97) of the included articles.

More than a single category of participants was identified in 19.6% of the articles analyzed (Table 2). Two categories of participants were targeted in 17.5% of the articles included (17/97) and three categories in 2.1% of articles analyzed (2/97).

#### 3.1.2. Involvement in the Included Articles

**Figure 3:**
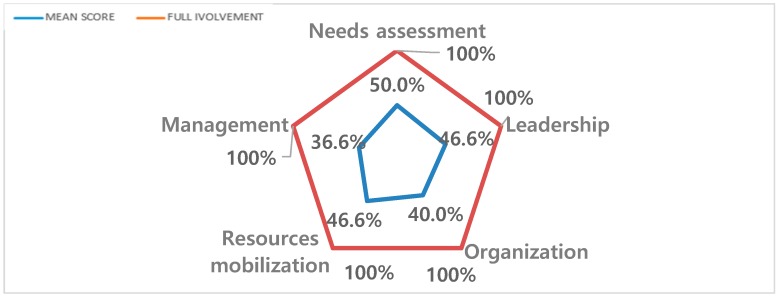
Level of involvement in included articles.

Each included article was classified into the group of interventions with the participants’ involvement when the sum of the score of Rifkin’s five items was greater than 5 (Table 1), otherwise in the group of non-involvement interventions. Out of 97 health interventions (Figure 2) analyzed, 19 (19.6% of the included articles) met the criterion of intervention with involvement. During the analysis, the articles’ mean score of involvement was about 35.3 % (5.3 points) for all the articles included and 44% for intervention with public involvement (Figure 3). Moreover, 11 of the included articles were rated above 5 for a single item of the Rifkin framework.

#### 3.1.3. Content of the Involvement in the Included Articles

Participants of interventions included nurses and doctors, residents, or family members. The content of participants’ involvement was related to the 19 articles analyzed, with a score over 5. The categories of participants of these nineteen articles were residents (five articles), nurses (10 articles), residents and nurses (two articles), residents and relatives (one article), and physicians and nurses (one article). The content of the involvement was described using Rifkin’s five factors: Needs assessment, leadership, organization, resource mobilization, and management.

● Involvement of Residents

To perform the needs assessment, some residents were asked to define their objectives regarding the health problem targeted by the planned intervention [16]. These objectives, considered as needs, were taken into account in the design and implementation of the intervention. Other authors reported that the training given to the participants was adapted to their expectations. This assessment was also conducted through preliminary studies using participants’ interviews [17] to take into account their preferences [18,19]. In addition, this assessment was performed by asking participants to identify their needs based on a pre-determined list of problems identified by the researchers. This list included frequently encountered problems in relation to the topic of the intervention [20]. Therefore, intervention proposals were based on participants’ choices of the most encountered problems of their own practices.

The second factor described in the involvement of participants was leadership. In this review, leadership was highlighted in different ways. As mentioned in an article, residents who developed skills at the training stage were called “mentors” [16]. They assisted their peers and other professionals in their facility during the intervention [21]. Another aspect of the interventions involving participants described in this review is the “resource mobilizations” factor.

● Involvement of Health Professionals

Researchers of an included article used statistics provided to nurses according to their own health situation in relation to an identical population to inform needs assessment [22]. In addition, discussion meetings with the nurses were a way for researchers to carry out a needs assessment for the design of the intervention [23]. Moreover, the collaboration with peers over the type of staff targeted by the intervention [24] or surveys [25] was used as a needs assessment strategy. Concerning the leadership factor, staff members who performed well in the observation phase were considered internal facilitators and trainers [26] in this intervention. In another intervention, a participant was designated by the staff as a point of contact for the study in all participating facilities [26]. Furthermore, a fourth study allowed skilled nurses to manage all stages of the intervention [27]. Finally, researchers considered the expertise of participants to enable their development of new skills in the daily work [28]. The health professionals were also thought of in the interventions as members of their organization [26]. The organization factor was illustrated by working with some health professionals participating in an intervention as facilitators or interlocutors [29] for the researchers. Finally, the inclusion of nurses as trainers with their peers [28] and the organization of specific meetings or video chat sessions during certain interventions [24] were strategies used by other researchers as means of mobilizing resources.

### 3.2. The Questionnaire

**Figure 4:**
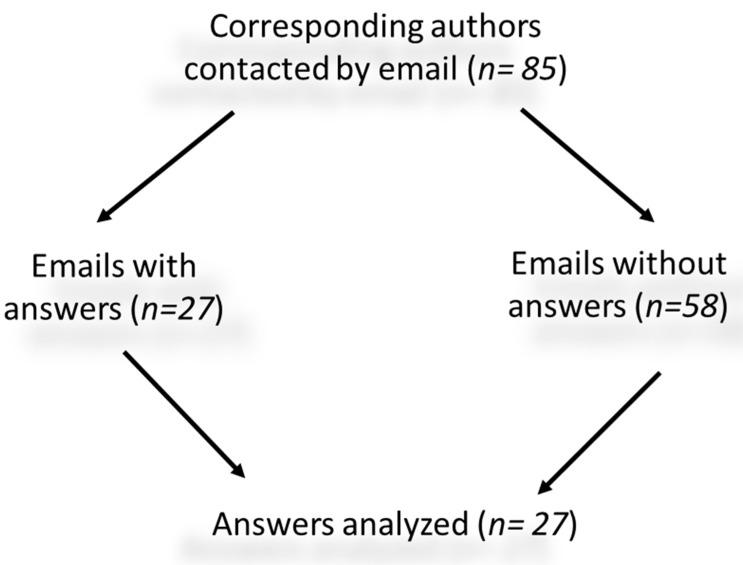
Questionnaires analyzed.

Eighty-five questionnaires were emailed to 85 corresponding authors (Figure 4). We obtained a response rate of 31.8% (27 articles out of 85). Four of the 27 articles for which a response was obtained met the criterion of public involvement in the intervention (score >5). These articles covered four topics: Loneliness (one article), the quality of nursing home care (two articles), and the hospitalization of residents (one article). We asked the corresponding authors to answer this question: “Did you involve the participants of your intervention in its design or management and how did you do it?”

Twenty-three corresponding authors who responded to the questionnaire confirmed the non-involvement of participants. Verbatim responses are presented in Table 3.

The involvement of participants was not a common practice of research during the implementation period of some interventions of this review (Box 1, Corresponding Author A). In other cases, authors confirmed non-involvement through brief statements as questionnaire responses (Box 1, Corresponding Author B). In addition, some interventions were designed and managed by the researchers or a third party (Box 1, Corresponding Author C). Otherwise, researchers designed or managed some of the interventions themselves (Box 1, Corresponding Author D).

Despite the high number of responses without the involvement of participants, other researchers were aware of participants’ involvement in health interventions and implemented their intervention by putting it in practice. Thus, four authors who responded to the questionnaire confirmed involvement of recipients, as was observed in the systematic review. Participants’ involvement was performed by designing the interventions’ tools with both researchers and participants or in another study, including the people studied in the research team (Box 1, Corresponding Author E).

In some interventions, authors illustrated the participants’ involvement by taking into account their opinion through feedback and try-out focus groups (Box 1, Corresponding Authors F, G, and H).

## 4. Discussion

The importance of involving participants in the different stages of research is of interest worldwide [30]. To our knowledge, no studies have been conducted to describe the involvement of residents and their relatives, nurses, and doctors in the design, development, and/or implementation of interventions in geriatric institutions. Therefore, we carried out a literature review to thoroughly describe this involvement. In particular, the present study described the extent and type of involvement implemented in 97 intervention studies targeting nurses, physicians, residents, or their relatives. These interventions were implemented in nursing homes where doctors who prescribed were most often off-site prescribers [31]. This explains the fact that nurses were the most reported category of participants in the selected interventions. The second most reported category of participants were residents. Nevertheless, residents and nurses were involved in various ways in the selected intervention studies.

### 4.1. Description of the Participants’ Involvement in the Health Interventions Analyzed

In this review, we observed that in interventions in geriatric facilities, involvement was rare (low prevalence) and its level was low. This is noteworthy since the importance and benefits highlighted in involving the public have been demonstrated [32] and stated by corresponding authors who completed the questionnaire.

Various reasons may explain this low prevalence and low level of involvement [33]. Firstly, the characteristics of the targeted participants (Frail residents, nurses and their workload) and the important challenges to involve them in the practice [34,35] may explain this low prevalence. Residents in nursing homes are often considered to be a population subgroup that requires additional time to be recruited and to obtain meaningful consent [36]. Thus, our results are consistent with another study that found that residents’ involvement in research within nursing homes was less well developed [36] than the others categories of the population.

An additional finding of this study was the fact that involvement was partially implemented when it existed, i.e., when not all the factors identified by the Rifkin analytical framework were fulfilled. Indeed, in many interventions, involvement was limited to a single Rifkin factor. Thus, the needs assessment was the most prevalent single factor implemented. This can be explained by the fact that needs assessment is fundamental when designing health interventions, which makes the content of the intervention more relevant to the issues inherent to the needs of the targeted population [37]. The involvement of participants at this stage of the intervention prior to design and implementation should generate a greater interest among these participants. Their interest would be linked to considering their real needs and expectations in terms of training and/or education [38]. However, it should be noted that not all interventions of this review involved participants in this step. The consequence of such a practice may be two-fold. On the one hand, the intervention may appear out of context with regard to the setting where it was implemented or to participants for whom it is delivered. On the other hand, without the needs assessment step, the achievement of the participants’ interest and the effect of the intervention would be limited. The partial involvement consists only of raising no impaired residents’ leadership by considering them as humans’ resources for the interventions. Leadership was an essential aspect of involvement in the articles included. Some authors perceived it as a means of receiving good quality care in in health care facilities [39]. According to some authors, leadership can contribute a key element to predict the sustainability of interventions [40]. Therefore, strengthening the leadership is a means of addressing the purposes of interventions that aim to improve the delivery of care and the quality of life of residents. In this review, leadership was illustrated by considering participants as internal resources for motivation and peer training. Thus, the focus on leadership as a facilitator contributes to ensuring that peers remain committed to the goals and values of an organization or action [41].

This review found that older people and nurses had relatively small incursions into the research process and researchers’ opinions continued to dominate [42,43]. These findings suggest a consumerist model of involvement where people have to make choices within predetermined interventions defined by researchers and implementers [44]. A second idea that emerged from this review is the fact that involvement could be seen as tokenistic [45]. In fact, only one aspect of involvement was frequently implemented. In brief, the results of this study underscore the challenges of involvement implementation in geriatric institutions and few changes in the practices, recommending a switch from the consumerist model or symbolic involvement to real and active involvement [46]. Therefore, adapted strategies are needed to raise the level of participants’ involvement in geriatric facilities interventions.

### 4.2. Increase of Participants’ Involvement in Geriatric Institutions Health Interventions

Residents of geriatric institutions are most often affected by cognitive disorders [35]. In some cases, they have little interest in interventions from which they do not always expect health benefits [35]. Therefore, the feasibility and relevance of their active involvement in the design or implementation of interventions in geriatric facilities can be difficult. Certain conditions are required to support public involvement in geriatric facilities. Researchers could undertake activities relevant to residents with cognitive disorders and motivate their genuine interest in the intended intervention project [47]. Preliminary feasibility studies could be necessary prior to public involvement. Moreover, empowerment may be another level of the involvement of residents in health interventions. Some authors suggested that researchers must be invested in empowering older people to improve their benefits from educational interventions [14]. Therefore, they could decide in a second time to change their practices [48].

For health professionals with clinical skills, interventions of this review were often delivered to nurses in geriatric facilities, such as nursing homes. However, time constraints and the high turnover of nurses could have been barriers to their active involvement in many interventions [49,50]. Taking into account the specificities of the participants remains the main driving force behind involvement in geriatric health interventions. Therefore, researchers must focus on residents’ disabilities as well as nursing time constraints and turnover before designing these interventions. Involvement could include an assessment of real needs and leadership of participants for effective ownership and sustainability of interventions. To reduce the time constraints of professionals when implementing interventions, researchers need to take into account the time dedicated to the intervention. They could also use digital technologies, such as the internet and video recording, in a setting where human resources and time are very limited. These technologies can reduce time-consuming face-to-face interactions and provide responsive interventions regarding professionals’ schedules and virtual discussion forums.

### 4.3. Limitations of This Review

The present study has some limitations. One of the limitations is the relatively low response rate. Notably, no additional responses were received after the initiation of the survey data analysis. This response rate is in line with those found in other email survey [51]. Furthermore, although we used an analytical framework, accurately assessing involvement in geriatric facilities remains challenging. In addition, some authors no longer belonged to the institutions from which they had published the article and their new email addresses were not found. Another limitation is the language of the included articles, despite the small number of articles in other languages. Finally, the time period of the study collection was limited; therefore, our review may not represent the average literature on the topic in the past decade.

## 5. Conclusions

The involvement of participants in health interventions is of real interest, as it allows empowerment [52,53]. Concomitantly, the population is aging and, in turn, health interventions among the geriatric population will be required more and more. However, involvement of participants in these settings is still uncommon and often consists of a consumerist involvement model, as shown in this review. Involvement in geriatric institutions still remains a challenge [14,49]. To facilitate its implementation, researchers should adopt attitudes that reflect the type of participants. Assessments of the feasibility of intended interventions and targeted participants could also help in the involvement process. Another possibility would be to consider residents’ living conditions or professional health conditions as well as the organizational constraints of the geriatric facilities. In short, researchers must try to find various solutions, such as an increase in participants’ interests in interventions [14], and involve them secondarily in the design and implementation of the interventions. Such strategies may facilitate participants’ ownership of these interventions and may contribute to improved sustainability and effectiveness. These approaches may also help to reduce health inequalities among older people in geriatric institutions.

## Figures and Tables

**Table 1:** Basis of the involvement assessment.

Points | Items	Needs Assessment	Leadership	Organization	Resources Mobilization	Management
1 point	Researchers are the unique decision makers about the interventions				
2 points	Both researchers and end-users are involved in the item				
3 points	End-users are the main decision makers for all items				
Total of the five items	XXX				

**Table 2:** Interventions targeting more than one category of participants.

Participants	Number of Articles
Residents + nurses	4 articles
Residents + relatives	2 articles
Relatives + nurses + physicians	1 article
Residents + nurses+ physicians	1 article
Physicians + nurses	11 articles
TOTAL	19 articles

**Table 3:** Answers of corresponding authors to the open-ended questionnaire.

Respondents	Verbatim
Corresponding Author A from Germany	“The study took part between 2004 and 2010. The intervention was developed in 2006 and 2007. At this time, the involvement of end users and participants in developing interventions was not common in Germany. So, we did not involve any residents and nurses in developing the intervention. But some of our team members were nurses (I for example).”
Corresponding Author B from Switzerland	“Thanks for your mail and interest. We did not involve older people in the design and management of the intervention.”
Corresponding Author C from USA	“To quickly answer your questions, nursing staff were not involved in the design of the intervention—a third party company designed and implemented the program. The third party managed the training and implementation for xx years.”
Corresponding Author D from the Netherlands	“Staff of research did the education component of the interventions (teaching, newsletter, etc.)”
Corresponding Author E from the Netherlands	“There was a lot of work and consultations that were done in the design and creation of the videos, including long-term care staff, administrators, and people living with HIV. In terms of the brief evaluation to inform the implementation and dissemination of the videos, we had a member of our team (who is an author on the paper) who was a nursing home administrator. He was involved in the design of the evaluation. In terms of implementing the training at the different homes as discussed in the paper we reached out to the individual nursing homes and worked with their staff to help facilitate the education sessions.”
Corresponding Author F from the Netherlands	“Yes, dual sensory impaired older adults, nurses, and care professionals (specialized in dual sensory impairment) were involved in the choice of the primary outcome measure and in the development of the intervention. To determine the primary outcome of the intervention, a focus group of dual sensory impaired older adults and their care professionals was asked ‘to identify the key aim of the psychosocial intervention’. Also, the group received and examined a variety of outcome measures. After discussion, they advised that social participation should be assigned as the primary outcome of the intervention. We performed a try-out in two different care facilities, discussed and collected the reactions and advices of the older adults and nurses, and adjusted the intervention.”
Corresponding Author G from the Netherlands	“The intervention comprised the … tools (development described by …) and improvement suggestions based on direct family feedback.”
Corresponding Author H from South Korea	“The health coaching program is that recipients actively join the intervention and finally set their own goals. Therefore, recipients involved the intervention. Qualified coaches formed rapports with recipients. Coaches gave recipients individual or group education. During the training, the coaches kept encouraging participants to set their own goals. It is an important intervention in the health coaching program to set and practice goals by oneself. Facilities provided a place for group training.”

## Supplementary Materials

The following are available online at https://www.mdpi.com/1660-4601/16/16/2812/s1. The data that support the findings of this systematic review can be found in the additional supporting files: Table S1: Query combinations, Table S2: Critical appraisal: Available online: https://drive.google.com/file/d/1lYg9gcG0T6QjzHMuly7iFTAti6MYL49D/view.
