# Supplementary material for: End-Users and Caregivers’ Involvement in Health Interventional Research Carried Out in Geriatric Facilities: A Systematic Review
Source: Int J Environ Res Public Health. 2019 Aug 7;16(16):2812. doi: 10.3390/ijerph16162812 (PMC6719053; doi:10.3390/ijerph16162812)
Supplement: Supplementary file 1 [file ijerph-16-02812-s001.pdf]

Table S1: Query combinations

- (Nursing home*[Ti] AND (education*[Ti] OR intervention*[Ti] OR stewardship*[Ti] OR training*[Ti] OR learning*[Ti])) OR
- (long-term care [Ti] AND (education*[Ti] OR intervention*[Ti] OR stewardship*[Ti] OR training*[Ti] OR learning*[Ti])) OR
- (residential aged*[Ti] AND (education*[Ti] OR intervention*[Ti] OR stewardship*[Ti] OR training*[Ti] OR learning*[Ti])) OR
- (residential care home*[Ti] AND (education*[Ti] OR intervention*[Ti] OR stewardship*[Ti] OR training*[Ti] OR learning*[Ti])) OR
- (care home*[Ti] AND (education*[Ti] OR intervention*[Ti] OR stewardship*[Ti] OR training*[Ti] OR learning*[Ti]))
